# The anti-apoptotic ubiquitin conjugating enzyme BIRC6/BRUCE regulates autophagosome-lysosome fusion

**DOI:** 10.1080/15548627.2018.1471311

**Published:** 2018-07-20

**Authors:** Fumiyo Ikeda

**Affiliations:** Institute of Molecular Biotechnology (IMBA), Vienna Biocenter (VBC), Vienna, Austria

**Keywords:** Autophagosome-lysosome fusion, BRUCE, IAP family, macroautophagy, siRNA screen, ubiquitin enzyme

## Abstract

The Inhibitor of Apoptosis (IAP) family member, Baculoviral IAP Repeat Containing 6 (BIRC6)/BRUCE is a ubiquitin conjugating E2 enzyme and a well-established anti-apoptosis regulator. However, its role in mammalian autophagy has not been shown. We identified BIRC6 as an important positive regulator of macroautophagy/autophagy by performing an siRNA screen targeting enzymes in the ubiquitin pathway. Compared to wild-type cells, BIRC6-deficient cells show accumulation of lipidated LC3B both at basal and starved conditions. Furthermore, BIRC6 deficiency blocks starvation-induced autophagic flux monitored by a tandem fluorescent autophagy sensor, mCherry-GFP-LC3B. Most strikingly, fusion of autophagosomes and lysosomes is blocked in BIRC6-deficient cells. BIRC6 colocalizes with the lysosomal protein LAMP2 in cells, and biochemically interacts with STX17 (syntaxin 17), which is a marker for completed autophagosomes. These data collectively suggest that BIRC6 bridges lysosomes and autophagosomes by interacting with these proteins. Because a deletion mutant of *BIRC6* lacking the UBC domain partially rescues the autophagosome-lysosome fusion defect in BIRC6-deficient cells, a role of BIRC6 in this event is independent of its E2 catalytic activity.

Ubiquitination is a part of the regulatory mechanisms of autophagy, especially in selective types of autophagy such as mitophagy, xenophagy and aggrephagy. In selective autophagy, ubiquitinated cargos are brought to phagophores, the precursors to autophagosomes, via autophagy receptors such as SQSTM1/p62, bridging cargos and autophagic MAP1LC3/LC3 proteins on the phagophore. However, in general, only a little is understood about how enzymes in the ubiquitin pathway regulate the autophagic machinery.

To better understand a link between the ubiquitin system and autophagy, we aimed to identify enzymes in the ubiquitin pathway that regulate starvation-induced autophagy [1]. To this end, we generated an shRNA library targeting more than 700 genes of enzymes in the ubiquitin pathway and known essential components of the autophagy machinery as positive controls. At the same time, we established a monoclonal mouse embryonic fibroblast (MEF) line stably expressing a pH-dependent autophagic sensor, mCherry-GFP-LC3B. We monitored loss of GFP signal upon amino acid starvation, which mediates the delivery of LC3B-positive vesicles into acidic autolysosomes. Following introduction of the home-made shRNA library in mCherry-GFP-LC3B MEFs, we starved the cells, sorted them by FACS based on GFP-low versus GFP-high signals and identified shRNA in each gate by next-generation sequencing. From this screen with a stringent cut-off setting, we identified 9 genes (*Atg7, Atg16l1, Birc6, Rb1cc1, Tsg101, Atg12, Sqstm1, Nedd8* and *Atg9a*), 5 of which are *Atg* (autophagy-related) genes implying that the screen was successful. Most importantly, we identified a previously unknown mammalian-autophagy regulator, BIRC6.

Baculoviral IAP Repeat Containing 6 (BIRC6)/BRUCE/Apollon is an inhibitor of apoptosis (IAP) family member and has a ubiquitin-conjugating E2 enzyme domain (UBC). BIRC6 is exceptionally large (more than 500 kDa) and is the only IAP family member to contain a UBC domain, whereas other members IAP1, IAP2 and XIAP are RING-type ubiquitin E3 ligases. Like other family members, BIRC6 inhibits apoptosis by degrading apoptosis regulators through the ubiquitin-proteasome system (UPS). In flies, Bruce has been implicated in autophagy and was suggested to be a negative regulator of autophagy. However, its role in mammalian autophagy has not been studied before.

In BIRC6-deficient MEFs stably expressing mCherry-GFP-LC3B, we confirmed that loss of the starvation-induced GFP signal is impaired. Furthermore, both lipidated and non-lipidated forms of LC3B, GABARAP and GABARAPL1 are highly accumulated in BIRC6-deficient cells in comparison to wild-type cells. Starvation induces formation of LC3B-positive autophagosomes as well as mature autophagosomes positive for STX17 in both wild-type and BIRC6-deficient cells. These data collectively suggest that BIRC6 regulates autophagic flux without affecting formation and maturation of autophagosomes. We observed that starvation-induced autolysosome formation is drastically disturbed in BIRC6-deficient cells, and numbers of STX17-positive mature autophagosomes are highly increased at the basal condition in these cells, further supporting the idea that BIRC6 regulates autophagosome-lysosome fusion.

Mechanistically, BIRC6 interacts with STX17 based on GST affinity-isolation assay, and colocalizes with the lysosomal protein LAMP2 as determined by confocal microscopy. Electron microscopy analysis suggests that transport of autophagosomes to lysosomes is not affected by BIRC6-deficiency. Based on these results, our working hypothesis is that BIRC6 bridges mature autophagosomes and lysosomes via STX17 and LAMP2, respectively, to promote their fusion (Figure 1).

**Figure 1.**
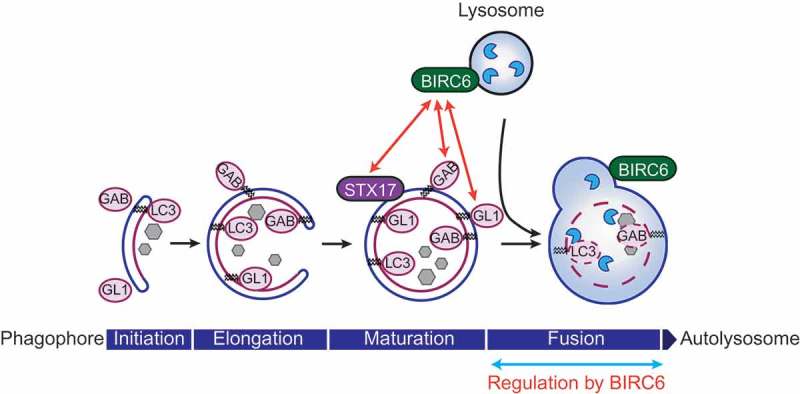
BIRC6/BRUCE regulates autophagosome-lysosome fusion. BIRC6 is localized at lysosomal/autolysosomal membranes in cells. BIRC6 interacts with GABARAP (GAB) and GABARAPL1 (GL1) among LC3 family members, as well as STX17, which specifically localizes on mature autophagosomes. Because BIRC6-deficient cells are defective in autophagosome-lysosome fusion, we propose that BIRC6 regulates the fusion step during macroautophagy induction.

Interestingly, BIRC6 selectively interacts with GABARAP and GABARAPL1 among the 6 LC3 family members (LC3A, LC3B, LC3C, GABARAP, GABARAPL1 and GABARAPL2) we analyzed. LC3 family proteins have homologous structures. It is not yet entirely clear if each member has unique and non-redundant functions in the regulation of autophagy; however, recent studies have suggested that GABARAPs have distinct functions from other members. For example, GABARAP proteins are more important than other LC3 proteins in autophagosome-lysosome fusion, and a pool of GABARAP resides on a centrosome region, which traffics from the Golgi to form autophagosomes. BIRC6 was also shown to localize with trans Golgi network markers in mammalian cells. Therefore, a BIRC6-GABARAP interaction might also be involved in autophagic trafficking processes.

It would be an interesting question whether autophagy regulation by BIRC6 is totally independent from its role in the anti-apoptosis pathway. BIRC6 is associated with cancer in humans, and both cell death and autophagy are highly relevant in cancer biology. Thereby, understanding more detailed molecular mechanisms of how BIRC6 regulates cell death and autophagy may contribute to the development of therapies against cancer in the future.
